# Metastatic Death Based on Presenting Features and Treatment for Advanced Intraocular Retinoblastoma: A Multicenter Registry-Based Study

**DOI:** 10.1016/j.ophtha.2022.04.022

**Published:** 2022-04-30

**Authors:** Ankit Singh Tomar, Paul T. Finger, Brenda Gallie, Tero T. Kivelä, Ashwin Mallipatna, Chengyue Zhang, Junyang Zhao, Matthew W. Wilson, Rachel C. Brennan, Michala Burges, Jonathan Kim, Jesse L. Berry, Rima Jubran, Vikas Khetan, Suganeswari Ganesan, Andrey Yarovoy, Vera Yarovaya, Elena Kotova, Denis Volodin, Yacoub A. Yousef, Kalle Nummi, Tatiana L. Ushakova, Olga V. Yugay, Vladimir G. Polyakov, Marco A. Ramirez-Ortiz, Elizabeth Esparza-Aguiar, Guillermo Chantada, Paula Schaiquevich, Adriana Fandino, Jason C. Yam, Winnie W. Lau, Carol P. Lam, Phillipa Sharwood, Sonia Moorthy, Quah Boon Long, Vera Adobea Essuman, Lorna A. Renner, Ekaterina Semenova, Jaume Català-Mora, Genoveva Correa-Llano, Elisa Carreras

**Affiliations:** 1 Department of Ocular Tumor, Orbital Disease, and Ophthalmic Radiation Therapy, The New York Eye Cancer Center, New York, New York; 2 The Eye Cancer Clinic, Princess Margaret Cancer Centre, and Department of Ophthalmology and Vision Sciences, Hospital for Sick Children, Toronto, Canada; 3 Ocular Oncology Service, Department of Ophthalmology, University of Helsinki and Helsinki University Hospital, Helsinki, Finland; 4 Pediatric Oncology Center, Beijing Children’s Hospital, Beijing, China; 5 Department of Ophthalmology, Hamilton Eye Institute, University of Tennessee Health Science Center, College of Medicine, and Department of Oncology, St. Jude Children’s Research Hospital, Memphis, Tennessee; 6 USC Roski Eye Institute, Keck Medical School of the University of Southern California, Los Angeles, California; The Vision Center at Children’s Hospital Los Angeles, Los Angeles, California; 7 Department of Vitreoretina Services, Sankara Nethralaya, Chennai, Tamil Nadu, India; 8 Ocular Oncology Department, The S.N. Fyodorov Eye Microsurgery Federal State Institution, Moscow, Russian Federation; 9 Department of Surgery (Ophthalmology), King Hussein Cancer Center, Amman, Jordan; 10 SRI of Pediatric Oncology and Hematology of N.N. Blokhin National Medical Research Center Oncology of Russian Federation, Moscow, Russian Federation; 11 Medical Academy of Postgraduate Education, Moscow, Russian Federation; 12 Department of Ophthalmology, Hospital Infantil de México Federico Gómez, Mexico City, Mexico; 13 Precision Medicine Coordination Hospital JP Garrahan and CONICET, National Scientific and Technical Research Council, Buenos Aires, Argentina; 14 Ophthalmology Service Hospital JP Garrahan, Buenos Aires, Argentina; 15 Department of Ophthalmology and Visual Sciences, The Chinese University of Hong Kong, Hong Kong Eye Hospital, Kowloon, Hong Kong; 16 Save Sight Institute, Discipline of Ophthalmology, Sydney Medical School, University of Sydney, Sydney, Australia; 17 KK Women’s and Children’s Hospital, Singapore; 18 Ophthalmology Unit, Department of Surgery, University of Ghana Medical School, College of Health Sciences, University of Ghana, Accra, Ghana; 19 Department of Child Health, University of Ghana Medical School, College of Health Sciences, University of Ghana, Accra, Ghana; 20 Retinoblastoma Unit, Department of Ophthalmology, Hospital Sant Joan de Déu. Esplugues de Llobregat, Barcelona, Spain; 21 Hemato-Oncology service, Hospital Sant Joan de Déu, Barcelona, Spain; 22 Department of Ocular Oncology, Narayana Nethralaya Eye Hospital, Bangalore, India

**Keywords:** Advanced, AJCC, Chemotherapy, Enucleation, International, Metastasis, Multicenter, Registry, Retinoblastoma, Staging

## Abstract

**Purpose:**

To evaluate presenting features, tumor size, and treatment methods for risk of metastatic death due to advanced intraocular retinoblastoma (RB).

**Design:**

International, multicenter, registry-based retrospective case series.

**Participants:**

A total of 1841 patients with advanced RB.

**Methods:**

Advanced RB was defined by 8th edition American Joint Committee on Cancer (AJCC) categories cT2 and cT3 and new AJCC-Ophthalmic Oncology Task Force (OOTF) Size Groups (1: < 50% of globe volume, 2: > 50% but < 2/3, 3: > 2/3, and 4: diffuse infiltrating RB). Treatments were primary enucleation, systemic chemotherapy with secondary enucleation, and systemic chemotherapy with eye salvage.

**Main Outcome Measures:**

Metastatic death.

**Results:**

The 5-year Kaplan–Meier cumulative survival estimates by patient-level AJCC clinical subcategories were 98% for cT2a, 96% for cT2b, 88% for cT3a, 95% for cT3b, 92% for cT3c, 84% for cT3d, and 75% for cT3e RB. Survival estimates by treatment modality were 96% for primary enucleation, 89% for systemic chemotherapy and secondary enucleation, and 90% for systemic chemotherapy with eye salvage. Risk of metastatic mortality increased with increasing cT subcategory (*P* < 0.001). Cox proportional hazards regression analysis confirmed a higher risk of metastatic mortality in categories cT3c (glaucoma, hazard ratio [HR], 4.9; *P* = 0.011), cT3d (intraocular hemorrhage, HR, 14.0; *P* < 0.001), and cT3e (orbital cellulitis, HR, 19.6; *P* < 0.001) than in category cT2a and with systemic chemotherapy with secondary enucleation (HR, 3.3; *P* < 0.001) and eye salvage (HR, 4.9; *P* < 0.001) than with primary enucleation. The 5-year Kaplan–Meier cumulative survival estimates by AJCC-OOTF Size Groups 1 to 4 were 99%, 96%, 94%, and 83%, respectively. Mortality from metastatic RB increased with increasing Size Group (*P* < 0.001). Cox proportional hazards regression analysis revealed that patients with Size Group 3 (HR, 10.0; *P* = 0.002) and 4 (HR, 41.1; *P* < 0.001) had a greater risk of metastatic mortality than Size Group 1.

**Conclusions:**

The AJCC-RB cT2 and cT3 subcategories and size-based AJCC-OOTF Groups 3 (> 2/3 globe volume) and 4 (diffuse infiltrating RB) provided a robust stratification of clinical risk for metastatic death in advanced intraocular RB. Primary enucleation offered the highest survival rates for patients with advanced intraocular RB.

Cancer staging systems serve as an essential tool for defining tumor extent, planning management approaches, and assessing prognosis.^1,2^ A growing trend exists toward attempted conservative treatment of advanced intraocular retinoblastoma (RB).^3–5^ Thus, a classification system for RB should (as precisely as possible) predict which advanced RB eyes can be safely salvaged versus those at high risk of systemic metastases at diagnosis or local treatment failure with recurrent disease. In that RB is a clinical diagnosis, efforts have been made to identify clinical risk factors at presentation that would predict high-risk pathology and, thus, metastasis. Such features include neovascularization of the iris, increased intraocular pressure, glaucoma, buphthalmos, and intraocular hemorrhage.^6–11^ These clinical high-risk features are typically recorded during the initial diagnosis in advanced RB eyes when eye cancer specialists and affected families decide to pursue goals for primary treatment. The question often comes down to a choice to remove or preserve the eye. Throughout the world, staging systems are used as tools to help clinicians make clinical decisions.

The 8th edition American Joint Committee on Cancer (AJCC) RB staging system is both comprehensive and evidence based.^12^ It has achieved worldwide adoption by incorporating into the Union for International Cancer Control staging system and The College of American Pathologists’ Instruction Manuals.^12–14^

For example, at least 1 multicenter, international, registry-based analysis of 2190 patients demonstrated that the 8th edition AJCC’s RB system had more excellent utility and prognostic value for both globe and life salvage than prior classification systems.^15,16^

It is important to note that the 8th edition AJCC RB staging system uniquely stratifies clinical high-risk RB features within the cT2 and, especially, the cT3 categories. In contrast, prior classification systems lump the clinical features itemized in cT3 together in a single cluster, “group E.”^17,18^ It is confusing that the past definitions for group E were never standardized. For example, the International Classification for Retinoblastoma, also known as the Wills Eye Hospital (WEH) group E, includes any tumor > 50% of globe volume,^18^ whereas the International Intraocular Retinoblastoma Classification or Children’s Hospital of Los Angeles (CHLA) group E includes diffuse infiltrating RB with no size criteria.^17^ The AJCC 7th edition RB staging scored high risk to a tumor volume > 2/3 of the eye.^19^ Overall, this lack of standardization created confusion, prevented research meta-analyses, and risked poor treatment protocols for patient management.^20,21^

Two prior evaluations of RB presentation versus national income revealed that the most common global presentation was AJCC category cT3 and that stratifying cT3 clinical features at diagnosis might more accurately predict risk for metastatic disease.^22,23^ These examples demonstrate how accurate cancer staging can equip treating physicians to advise RB families on safe treatment choices. The scope of this study is to assess the risk of metastatic mortality based on presenting clinical features, intraocular tumor size, and treatment modalities.

## Methods

**Figure 1.**
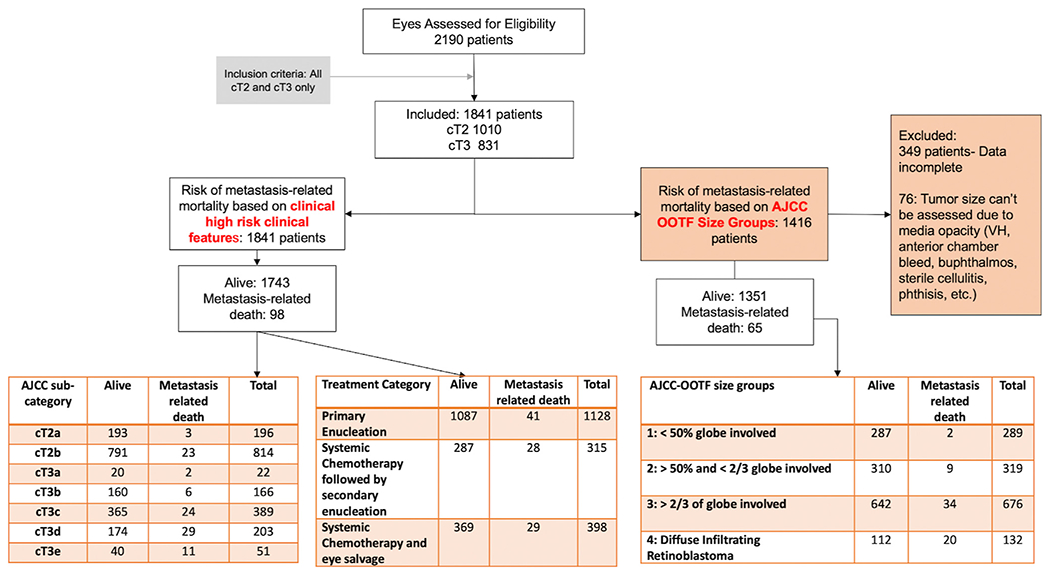
Consolidated Standards of Reporting Trials flow diagram of all patients with advanced retinoblastoma. AJCC = American Joint Committee on Cancer; OOTF = Ophthalmic Oncology Task Force; VT = vitreous hemorrhage.

The pooled registry data used in this study were derived from 2190 patients enrolled across 18 RB centers from 13 countries on 6 continents (Fig 1). The data were analyzed for mortality from advanced intraocular RB, defined as AJCC clinical cT2 and cT3 categories. The patients were diagnosed between January 5, 2001, and December 31, 2013. All participating centers obtained internal institutional review board approval before retrospective medical record reviews and anonymized data entry into a secure online database. The Princess Margaret Cancer Center determined, and all centers agreed, that individual patient consent was not required because no patient identifiers were collected in this retrospective study. The AJCC Ophthalmic Oncology Task Force (OOTF) committee members developed the data field questions used in this study. Our internet database and security methods have been described in our prior publications.^15,16,22^ This study was conducted to adhere to the Declaration of Helsinki and the Health Insurance Portability and Accountability Act of 1996.

### Definitions

The participating centers were ophthalmic oncology subspecialty sites, and the patients were diagnosed and treated as per best practices defined by each center. Clinical records were reviewed, and data collected included demographic and clinical information comprising size and location of the intraocular tumor and presence of phthisis or prephthisis bulbi, anterior segment tumor invasion, glaucoma, iris neovascularization, buphthalmos, hyphema, vitreous hemorrhage, or aseptic orbital cellulitis. For bilateral RB, by AJCC convention, the worse-eye tumor category was taken to be the patient-level clinical (cT) category for survival analysis. Likewise, the treatment for the worse category eye in bilateral RB was attributed to patients and used for treatment modality analysis.

Outcome data included the occurrence and the date of detection of metastasis and site of metastasis. In addition, the final patient outcome (alive without metastasis, alive with metastasis, dead with metastasis, dead of other causes, or lost to follow-up), date of the last follow-up, and duration of follow-up were noted. Patients who developed central nervous system metastasis and were then lost to follow-up were considered deceased (included in the metastasis-related mortality analysis). Patients whose treatment was discontinued by request of their guardians and were then lost to ophthalmic follow-up but eventually recorded as having died of RB were also included as deaths in the analysis of metastasis-related mortality. All other nonmetastasis-related deaths were censored observations in the analysis (and were thus not modeled as competing risk events).

Primary RB tumor extent was defined according to the 8th edition AJCC Cancer Staging Manual.^12^ High-risk clinical features (Table 1) for advanced RB were stratified in cT2 and cT3 subcategories: notable retinal detachment with risk of subretinal tumor cells (cT2a); seeding (cT2b); phthisis bulbi (cT3a); anterior segment tumor invasion (cT3b); rubeosis iridis with neovascular glaucoma (cT3c); hyphema, massive vitreous hemorrhage, or both (cT3d); and aseptic orbital cellulitis (cT3e). Data were available for all subcategories except the involvement of pars plana and ciliary body (a component in cT3b). In bilateral RB, the worst category eye was taken to represent the patient, as per AJCC protocol.

### AJCC-OOTF Size Group Definitions

Multiple criteria for intraocular tumor size have been used to estimate the risk of treatment failure. The AJCC 7th edition RB staging system used a 2/3 fill of the ocular volume. The WEH used tumor filling > 50% of globe volume to define group E, and the CHLA defined group E as diffuse infiltrating RB.^17–19^ The presence of diffuse intraretinal and vitreal growth without a defined tumoral mass was Ashtons’ (1958) original definition of diffuse infiltrating RB.^17,24,25^ Considering these criteria and the paucity of foundational medical evidence, the AJCC-OOTF divided intraocular tumor size into the following 4 groups to study their risk for metastasis-related death:

Exclusion criteria for this study included missing or inconsistent key variables: clinical variables essential for RB classification (tumor location, size, extent), treatment data (date and type of treatment), and missing outcome data. The AJCC cT1 cases were excluded because they were treated with globe-conserving therapies, whereas cT4 eyes were excluded because overt orbital RB was not amenable to eye salvage.

The aim of this work is to analyze the risk of metastatic death in advanced RB based on presenting features, tumor size, and treatment. A companion study of high-risk pathology as associated with initial clinical features is published as a separate, although complementary, work.^26^

**AJCC-OOTF Size Groups**

1. < 50% of globe volume involved;
2. > 50% but < 2/3 of globe volume involved;
3. > 2/3 of globe volume filled with tumor;
4. Diffuse infiltrating RB.

### Statistical Analysis

The data were summarized per the AJCC 8th edition RB staging system and AJCC-OOTF Size Groups. The median, range, and interquartile range (IQR) were used to describe continuous variables, and frequencies and proportions were given for categorical variables. Kaplan–Meier plots, log-rank test for trend, and Cox proportional hazards regression models were used to test if cT2 and cT3 subcategories and Size Groups were independently related to metastasis-related mortality. The statistical analysis was performed using SPSS (version 26.0, IBM). Statistical significance was set at *P* < 0.0.

## Results

### Demographic and Clinical Features

The median age at diagnosis of the 1841 patients who belonged to the patient-level, that is, worse-eye, clinical cT2, or the cT3, AJCC category of anatomic extent (54.9% and 45.1% of patients, respectively), was 19.0 months (mean, 22.8; standard deviation [SD], 20.8; IQR, 9–30), and median follow-up was 43.0 months (mean, 52.8; SD, 41.7; IQR, 19–78). Median age at diagnosis for cT2 patients was 16.0 months (mean, 20.67; SD, 21.02; IQR, 8–29), which was significantly different (*P* < 0.001) from cT3 patients, which was 22.0 months (mean, 25.36; SD, 20.23; IQR, 12–33).

The patient-level subcategory was 10.6% of patients cT2a, 44.2% of cT2b, and cT3a-e in 1.2% (cT3a, phthisis), 9.0% (cT3b, anterior chamber involvement), 21.1% (cT3c, glaucoma), 11.0% (cT3d, intraocular hemorrhage), and 2.8% (cT3e, orbital cellulitis) of patients, respectively (Table 2).

The tumor size data were available for 1416 patients (76%): AJCC-OOTF Size Group 1 (< 50% of volume), 289 (20.4%); Size Group 2 (> 50% but < 2/3 of volume), 319 (22.5%); Size Group 3 (> 2/3 of volume), 676 (47.7%); and Size Group 4 (diffuse infiltrating RB), 132 (9.3%, Table 2).

### Treatment Outcomes

Of the 1841 patients with advanced RB, 1128 (61.3%) were treated with primary enucleation, and 713 (38.7%) were treated with systemic chemotherapy. Of the latter, 315 (44.2%) needed secondary enucleation, and 398 (55.8%) were salvaged (Table 2). The relationship between tumor laterality and preferred treatment modality was explored and found to be significant (chi-square = 169.334, *P* < 0.001) (Table S1 and Fig S1, available at http://www.aaojournal.org). Unilateral advanced RB was more commonly treated with primary enucleation than bilateral advanced RB. In addition, 106 patients received intra-arterial chemotherapy. Of these, 92 received subsequent systemic chemotherapy resulting in subsequent enucleation for 45 eyes, leaving 61 salvaged. Because of the small number of patients receiving intra-arterial chemotherapy in this cohort, a separate analysis was not performed for its influence on systemic outcome.

In this registry, 98 patients (5.3%) developed RB metastasis and eventually died of the disease. The median time from diagnosis to development of metastasis in 84 patients (with available data on duration to metastasis detection) was 9.5 months (mean, 13.5; SD, 13.2; IQR, 4.3–19.8). Of the 98 patients who developed metastasis, 3 (3%) were cT2a (with subretinal fluid), 23 (23%) were cT2b (with RB seeding), 2 (2%) were cT3a (phthisis), 6 (6%) were cT3b (anterior segment infiltration), 24 (24%) were cT3c (neovascular glaucoma), 29 (30%) were cT3d (intraocular hemorrhage), and 11 (11%) were cT3e (aseptic orbital cellulitis). Overall, 41 patients (3.6%) treated with primary enucleation died with metastasis, compared with 28 (8.9%) treated by systemic chemotherapy followed by secondary enucleation and 29 (7.3%) treated by systemic chemotherapy and eye salvage (Fig 1).

Of the 1416 patients with tumor size data, 65 (4.6%) developed metastatic disease and eventually died. Of these, 2 (3%) were in Size Group 1 (< 50% of volume), 9 (14%) were in Group 2 (> 50% but < 2/3 of volume), 34 (52%) were in Group 3 (> 2/3 of volume), and 20 (31%) were in Group 4 (diffuse infiltrative RB) (Fig 1, Table 2).

### Cumulative Proportion of Avoiding Metastatic Death by cTNM Category

**Figure 2.**
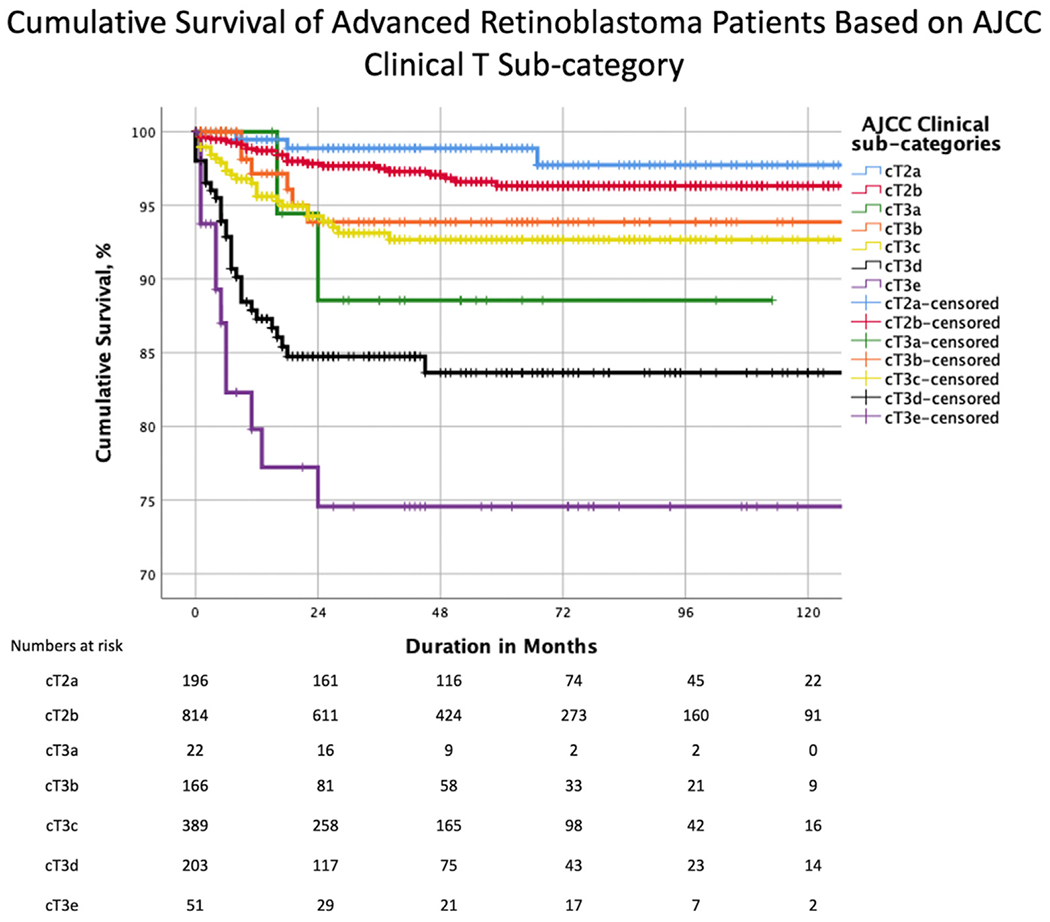
Kaplan–Meier curves showing cumulative survival estimates for patients with advanced retinoblastoma by American Joint Committee on Cancer (AJCC) clinical subcategory. T = tumor.

The 5-year Kaplan–Meier cumulative probabilities of survival by clinical AJCC categories were 98% for cT2a (subretinal fluid), 96% for cT2b (RB seeds), 88% for cT3a (phthisis), 95% for cT3b (anterior chamber involvement), 92% for cT3c (glaucoma), 84% for cT3d (intraocular hemorrhage), and 75% for cT3e (orbital cellulitis) (Table 3 and Fig 2). The 10-year survival was the same as the 5-year survival. Increasing tumor subcategory from cT2a to cT3e translated to an increased risk of metastasis-related death and shortening survival (*P* < 0.001, log-rank test for trend), except for cT3a, which was associated with a survival intermediate between cT3b and cT3d. However, the cT3a estimate is based on only 2 events (Fig 2).

Pairwise comparison after adjustment for multiple comparisons suggested comparable survival in cT2a and cT2b, a difference between cT2a and cT3d-e, cT2b and cT3c-e, cT3b and cT3e, and cT3c and cT3d-e (Table 3).

**Figure 3.**
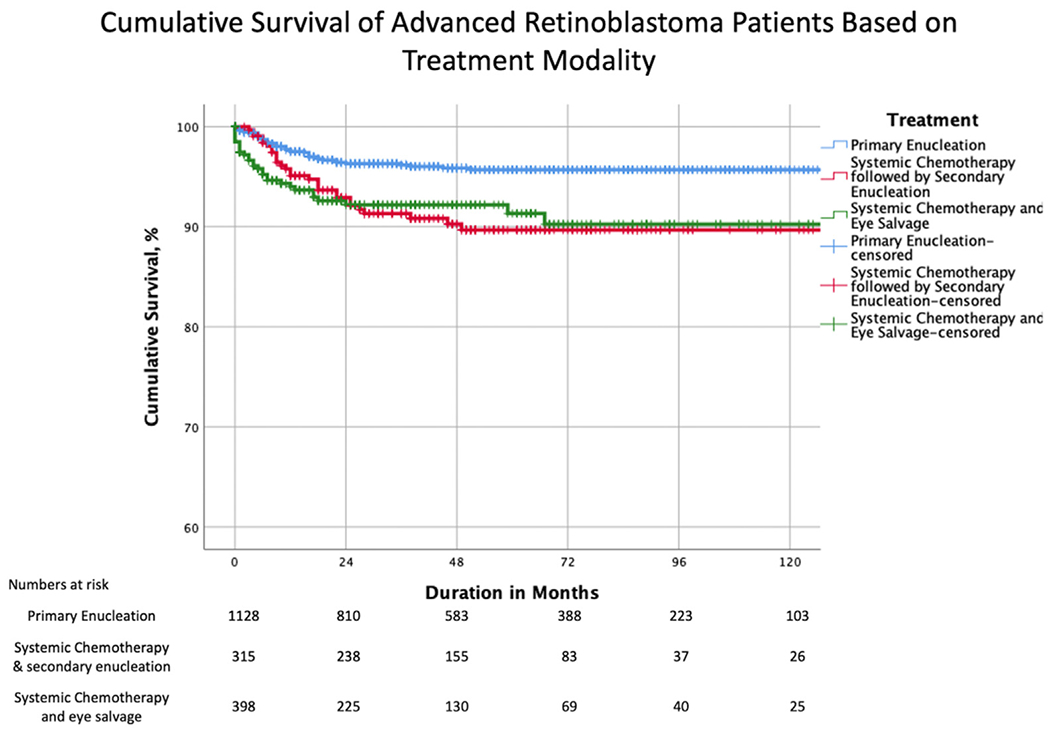
Kaplan–Meier curves showing cumulative survival estimates for patients with advanced retinoblastoma by treatment modality.

The 5-year Kaplan–Meier cumulative probability of patient survival by treatment modality was 96% for primary enucleation, 89% for systemic chemotherapy followed by secondary enucleation, and 90% for systemic chemotherapy with eye salvage (Table 3 and Fig 3). Therefore, the risk of metastatic mortality increased as the treatment shifted from primary enucleation of advanced RB to systemic chemotherapy with or without eye salvage (*P* < 0.001). A significant difference was noted in pairwise comparison (after adjustment for multiple comparisons) between primary enucleation and systemic chemotherapy (*P* < 0.001 for both secondary enucleation and eye salvage) but not between secondary enucleation and eye salvage (*P* = 0.951). No statistical difference was noted between metastatic deaths in unilateral and bilateral tumors (Fig S2, available at www.aaojournal.org, and Table 3). A comparison of cumulative survival by AJCC RB cT subcategory and treatment modality is shown in Table S2 and Figures S3 and S4 (available at http://www.aaojournal.org). The trend from primary enucleation to systemic chemotherapy translated to increasing risk of metastasis-related death and shortening survival in subcategories cT2a (subretinal fluid, *P* = 0.013, log-rank test for trend), cT2b (RB seeds, *P* = 0.025), cT3b (anterior chamber involvement, *P* = 0.029), cT3c (glaucoma, *P* < 0.001), and cT3e (orbital cellulitis, *P* = 0.004) but not for cT3a (phthisis, *P* = 0.382) or cT3d (intraocular hemorrhage, *P* = 0.147). The pairwise comparison revealed a significant difference in subcategory cT3c (glaucoma) between primary enucleation and systemic chemotherapy (*P* = 0.001 for secondary enucleation and *P* < 0.001 for eye salvage) and in subcategory cT3e (orbital cellulitis) between primary enucleation and systemic chemotherapy followed by eye salvage (*P* = 0.001) (Table S2, available at www.aaojournal.org).

The cT3a (phthisis) subcategory has few cases (n = 22), and the curve violates the proportional hazards assumption. Thus, the Cox proportional hazard ratio (HR) analysis was performed after excluding cT3a and showed that patients in cT3c (glaucoma, HR, 4.9; *P* = 0.011), cT3d (intraocular hemorrhage, HR, 14.0; *P* < 0.001) and cT3e (orbital cellulitis, HR, 19.6; *P* < 0.001) subcategories had a higher risk of metastasis-related death than those in cT2a (Table 4). Likewise, patients with advanced RB treated with systemic chemotherapy followed by secondary enucleation (HR, 3.3; *P* < 0.001) and chemotherapy and eye salvage (HR, 4.9; *P* < 0.001) had a higher risk of metastasis-related death than those treated with primary enucleation. Increasing age at presentation, 17.0 to 29.0 months (HR, 2.7; *P* = 0.002), and > 29.0 months (HR, 2.8; *P* = 0.002) were significantly related to risk of metastatic death compared with age at presentation < 8.0 months. However, the presence of heritable trait (H1, *P* = 0.116) was not significantly related to the risk of metastatic death when compared with the absence of heritable trait (H0).

### Cumulative Proportion of Avoiding Metastatic Death by Tumor Size

**Figure 4.**
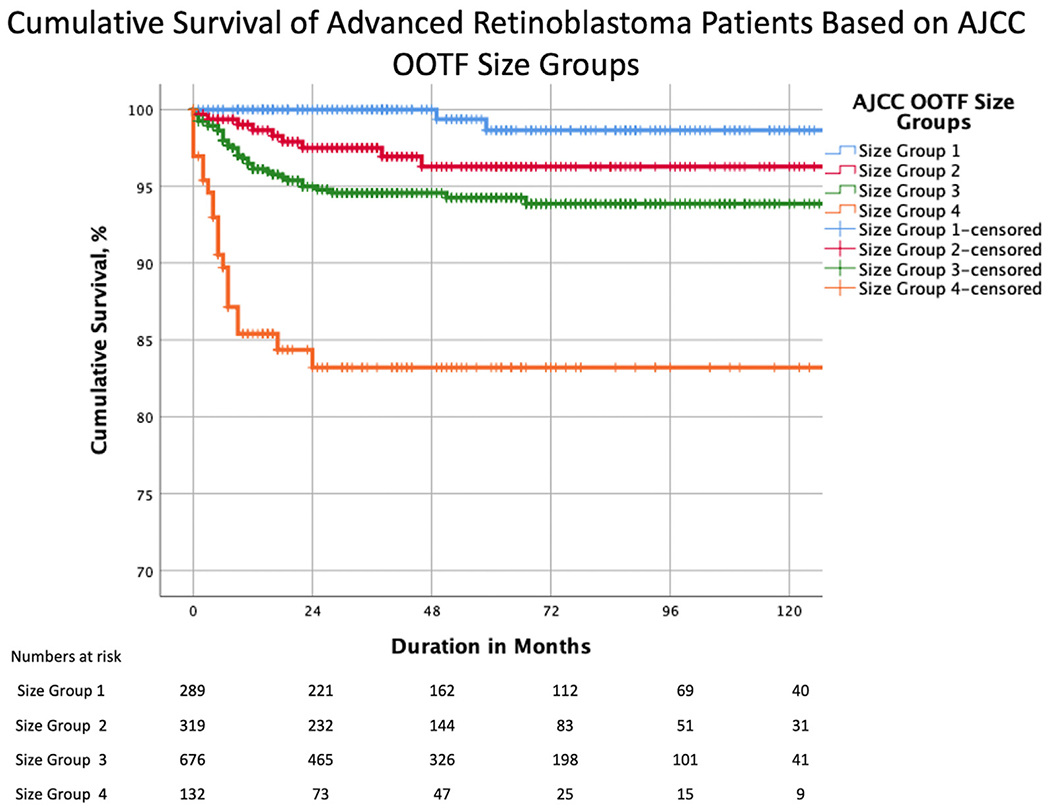
Kaplan–Meier curves showing cumulative survival estimates for patients with advanced retinoblastoma by American Joint Committee on Cancer (AJCC)-Ophthalmic Oncology Task Force (OOTF) Size Groups.

The 5-year Kaplan–Meier cumulative survival estimates by AJCC-OOTF Size Groups were 99%, 96%, 94%, and 83% for Size Groups 1, 2, 3, and 4, respectively (Table 5, Fig 3). Thus, the higher the Size Group, the greater the risk of metastasis-related death (*P* < 0.001, log-rank test for trend). Pairwise comparison after adjustment for multiple comparisons suggested a difference between Size Groups 1 and 3 and Size Groups 1 to 3 and 4 (Table 5, Fig 4). A comparison of cumulative survival between unilateral and bilateral RB based on tumor size has been elucidated in Table S3 and Figure S5 (available at www.aaojournal.org). Likewise, a comparison of cumulative survival by treatment modalities and AJCC-OOTF Size Groups is shown in Table S4 and Figure S6 (available at www.aaojournal.org). Increasing Size Group translated to increasing risk of metastasis-related death in primary enucleation and systemic chemotherapy with eye salvage (*P* < 0.001 for both, log-rank test for trend) and not for systemic chemotherapy followed by secondary enucleation (*P* = 0.114). Pairwise comparison after adjustment for multiple comparisons suggested a difference between Size Groups 1 to 3 and Size Group 4 in primary enucleation (*P* < 0.001 for all 3) and between Size Groups 1 and 3 to 4 (*P* = 0.001 and *P* < 0.001, respectively), and between Size Groups 2 and 3 to 4 (*P* = 0.003 and *P* < 0.001, respectively) in advanced RB receiving systemic chemotherapy with eye salvage (Table S4, available at www.aaojournal.org).

Cox proportional hazards regression analysis showed that patients with Size Group 3 (HR, 10.0; *P* = 0.002) and Size Group 4 (HR, 41.1; *P* < 0.001) had a greater risk of metastasis-related death than those with Size Group 1 (Table 6). Likewise, age at presentation 17.0 to 29.0 months (HR, 2.5; *P* = 0.032) and > 29.0 months (HR, 3.7, *P* = 0.003) had a greater risk of metastasis-related death than age at presentation < 8.0 months, respectively. In addition, the heritable trait (*P* = 0.052) was not significantly related to the risk of metastatic death compared with the absence of a heritable trait.

## Discussion

We analyzed data from a multicenter, international, internet-based registry to determine the risk of metastatic death from advanced intraocular RB at initial diagnosis. Our analysis was primarily based on clinical features as defined by 8th edition AJCC cT-categories, novel AJCC-OOTF Size Groups, and their treatment modalities. Increasing AJCC cT3 subcategories were associated with a higher risk of metastatic death (Fig 2). Specifically, we estimated a 4.9-fold risk for cT3c (neovascular glaucoma and buphthalmos), a 14.0-fold risk for cT3d (vitreous hemorrhage or hyphema), and a 19.6-fold risk for cT3e (aseptic orbital cellulitis) compared with cT2a. Increasing age at presentation and attempt at eye salvage by systemic chemotherapy were also significant risk factors for metastasis. We developed novel AJCC-OOTF Size Grouping with consideration to prior attempts.^17–19^ Increasing intraocular Size Group translated to an increased risk of metastatic death, with a 10.0-fold risk for AJCC-OOTF Size Group 3 (tumor involving > 2/3 of globe volume) and a 41.1-fold risk for Group 4 (diffuse infiltrating RB) compared with Group 1 (tumor involving < 50% of globe volume).

### Clinical High-risk Features

This study demonstrates that the primary ophthalmic evaluation is critical to guiding RB patient management. Various authors have analyzed the clinical features that may predict high-risk pathology and, consequently, risk of systemic metastasis. Glaucoma or buphthalmos was associated with high-risk pathology among 182 consecutive patients with unilateral RB treated with primary enucleation. In contrast, hyphema, orbital cellulitis, and diffuse infiltrative RB considered in the aggregate as “inflammatory eye” were not, and mortality was not analyzed.^6^ A more extensive study of 326 primarily enucleated eyes concluded that vitreous hemorrhage, hyphema, staphyloma, and orbital cellulitis were predictors of high-risk pathology.^7^ Therefore, despite significant literature identifying clinical high-risk features, only AJCC staging presently provides a prognosis-based risk stratification to support safe clinical decision making.^17,18^

This large, multicenter, international, data-sharing registry has provided statistically significant medical evidence to support findings that neovascular glaucoma or buphthalmos, intraocular hemorrhage, and aseptic cellulitis carry an increased risk of metastatic death. In addition, it revealed that treatment modality stratified analyses of these subcategories showed that cT3c (glaucoma) and cT3e (orbital cellulitis) offer a significantly different risk of metastasis-related death when treated by primary enucleation or salvage attempts with systemic chemotherapy. This finding might be due to intraocular pressure-related scleral thinning or inflammation-induced scleral breach (Table S2, available at www.aaojournal.org). In contrast, we found no difference in survival in the 3 treatment arms in subcategory cT3d (intraocular hemorrhage). A possible explanation could be that the bleeding obscures the true tumor extent. Lastly, we did not find a significant association of anterior segment involvement with metastatic death. This finding should be interpreted with caution because the registry’s clinical data did not account for specific involvement of the ciliary body and pars plana, which may have led to downstaging eyes that might have been otherwise assigned to the cT3b subcategory.

### Treatment Modalities

This study indicates that salvage attempts with systemic chemotherapy (irrespective of outcome: globe salvage or secondary enucleation) in advanced RB increase the risk of metastasis-related death compared with primary enucleation. That risk was 3.3-fold with systemic chemotherapy followed by secondary enucleation and 4.9-fold with chemotherapy and eye salvage. These results contrast with the clinical experience with RB in western countries, where timely follow-up and aggressive local therapies are feasible for the smallest recurrences. However, it is essential to note that increased patient age and advanced RB stage are more common presentations for patients in resource-poor countries.^22,23,27^ The pathology examination results are more likely delayed, incomplete, or absent in these areas.^28^ In addition, ultrasound biomicroscopy is less likely to be available.^11^ In those parts of the world, a reliable clinical high-risk feature stratification is the most valuable tool that can be used to assist clinicians in their decisions to prescribe adjuvant therapy.

### Tumor Laterality

Tumor laterality also affects the treatment strategy. For example, we found that unilateral advanced RB was more commonly treated with primary enucleation than bilateral disease. This strategy minimizes the metastatic risk in unilateral cases. However, our treatment modality and tumor laterality analysis did not significantly differ in metastasis-related deaths between unilateral and bilateral tumors in the same treatment arms (Fig S2, available at www.aaojournal.org, and Table 3). In addition, we used the heritable trait (H) category, which includes bilateral RB, trilateral RB, family history of RB, or molecular definition of constitutional RB1 gene mutation, as a factor in multivariable analysis (Tables 4 and 6), and the risk of metastasis-related death was not statistically significant. However, genetic testing was performed on a limited number of patients (n = 44, 2.1%) because of the timing of data collection and the local availability of genetic services. Thus, our H-status data may not represent the actual presence of heritable trait in patients with unilateral RB. Therefore, it could have led to underestimating its significance concerning metastatic disease. Now that the 8th edition AJCC staging system collects tumor, node, metastasis, and heritable trait data, future studies will be better able to explore the impact of heritable trait on mortality from RB and associated cancers.

### Age at Presentation

We found advanced age at presentation conferred a worse prognosis. This finding is supported by evidence from a recent study in which increasing age was correlated to high-risk genomic features.^29^

### Tumor Size

Discrepancies exist between tumor staging criteria for group E eyes between classification systems.^17–20^ This discrepancy was highlighted by our registry, where 31.3% were classified as group E per CHLA, whereas 61.6% of study eyes were group E using the WEH classification.^15^ To further complicate matters, the literature is filled with studies staging RB eyes by “international classification,” which fail to identify whether WEH or CHLA was used, creating perplexity for clinicians who manage RB.^14^ Kim et al^21^ have argued against the clinical size criteria for group E eyes and instead recommended that 1 uniform system be used for all advanced intraocular RB.

Our study found a higher than expected number of diffuse infiltrating RBs and that the corresponding AJCC-OOTF Size Group 4 was strongly associated with the risk of metastatic death. Specifically, the present study suggests a higher risk for metastasis when > 2/3 of the globe volume is filled with RB or diffuse infiltrating RB. Further analyses showed similar increased risk whether the advanced RB was treated with primary enucleation or systemic chemotherapy with eye salvage (Table S4, available at www.aaojournal.org). These findings suggest that > 2/3 of the globe volume is filled with RB, and diffuse infiltrating RB are reliable indicators for RB risk stratification and may be considered for further AJCC staging editions.

### Study Limitations and Strengths

The limitations of this study include its retrospective design and lack of data on pars plana and ciliary body involvement. In addition, our analysis is relevant only to the time point of initial diagnosis. It does not address the risk of metastatic death after treatments extended for any recurrent or refractory tumors. Because of the data collection period, too few patients treated with intra-arterial chemotherapy were included in our analysis. This prevented us from analyzing the impact of this treatment modality on systemic outcomes. In addition, the chemotherapy protocols were governed by the individual center’s treatment guidelines, and a chemotherapy protocol-based comparison was beyond the scope of this study. Finally, per the AJCC convention, our study was based on the worst eye, and a patient with bilateral RB carries a combined risk based on both eyes.

The strengths of this work include that it is an extensive, multicenter, global, registry-based analysis using a uniform staging system. There were a large number of patients from whom we could derive significant medical evidence to answer important clinical questions regarding a rare pediatric tumor.

## Conclusions

Evidence-based, multicenter collaborative research can be used to resolve the debate on clinical high-risk features and Size Group in managing RB. Specifically, this study’s pooled data analysis provided evidence of the following:

1. The 8th edition AJCC RB staging subcategories cT2 and cT3 allow stratification of clinical risk factors that can be used to predict metastasis-related mortality.
2. In the case of advanced RB, primary enucleation is a safer treatment option than attempts at eye salvage with systemic chemotherapy, especially in unilateral RB.
3. Advanced age at presentation confers a worse prognosis for metastatic disease.
4. The AJCC-OOTF Size Groups offer an opportunity to improve staging systems for RB.
5. The 8th edition AJCC classification for RB is derived from evidence-based data and international consensus. Herein, it has served as an effective tool to assess the clinical risk of metastatic death in advanced intraocular RB and to guide treatment planning.

## Supplementary Material

sup fig 1

sup fig 2

sup fig 3

sup fig 4

supp fig 5

supp fig 6

sup tab 1

sup tab 2

sup tab 3

sup tab 4

supp data

## Figures and Tables

**Table 1.** Definitions for 8th Edition AJCC Clinical Primary Tumor Staging of Retinoblastoma (cT)^11^

cTX		Unknown evidence of intraocular tumor
cT0		No evidence of intraocular tumor
cT1		Intraocular tumor(s) with subretinal fluid ≤5 mm from the base of any tumor
	cT1a	Tumors ≤ 3 mm and farther than 1.5 mm from the disc and fovea
	cT1b	Tumors > 3 mm or closer than 1.5 mm to the disc and fovea
cT2		Intraocular tumor(s) with retinal detachment, vitreous seeding, or subretinal seeding
	cT2a	Subretinal fluid > 5 mm from the base of any tumor
	cT2b	Tumors with vitreous seeding or subretinal seeding
cT3		Advanced intraocular tumor(s)
	cT3a	Phthisis or prephthisis bulbi
	cT3b	Tumor invasion of the pars plana, ciliary body, lens, zonules, iris, or anterior chamber
	cT3c	Raised intraocular pressure with neovascularization or buphthalmos
	cT3d	Hyphema or massive vitreous hemorrhage
	cT3e	Aseptic orbital cellulitis
cT4		Extraocular tumor(s) involving the orbit, including the optic nerve
	cT4a	Radiological evidence of retrobulbar optic nerve involvement or thickening of the optic nerve or involvement of the orbital tissues
	cT4b	Extraocular tumor clinically evident with proptosis and orbital mass

AJCC = American Joint Committee on Cancer; c = clinical; T = tumor.

**Table 2.** Patient-Level (Worse Eye) 8th Edition AJCC cT Category of Anatomic Extent in 1841 Patients with Retinoblastoma by Treatment Modality

			AJCC Clinical Tumor Category							
				
			*cT2a*	*cT2b*	*cT3a*	*cT3b*	*cT3c*	*cT3d*	*cT3e*	Total
Treatment modality	Primary enucleation	Count	110	410	13	123	284	161	27	1128
		% treatment modality	9.8	36.3	1.2	10.9	25.2	14.3	2.4	100.0
		% AJCC subcategory	56.1	50.4	59.1	74.1	73.0	79.3	52.9	61.3
		% total	6.0	22.3	0.7	6.7	15.4	8.7	1.5	61.3
	Systemic chemotherapy followed by secondary enucleation	Count	32	164	5	19	61	23	11	315
		% treatment modality	10.2	52.1	1.6	6.0	19.4	7.3	3.5	100.0
		% AJCC subcategory	16.3	20.1	22.7	11.4	15.7	11.3	21.6	17.1
		% total	1.7	8.9	0.3	1.0	3.3	1.2	0.6	17.1
	Systemic chemotherapy and eye salvage	Count	54	240	4	24	44	19	13	398
		% treatment modality	13.6	60.3	1.0	6.0	11.1	4.8	3.3	100.0
		% AJCC subcategory	27.6	29.5	18.2	14.5	11.3	9.4	25.5	21.6
		% total	2.9	13.0	0.2	1.3	2.4	1.0	0.7	21.6
Total		Count	196	814	22	166	389	203	51	1841
		% total	10.6	44.2	1.2	9.0	21.1	11.0	2.8	100.0

Patient-Level (Worse Eye) AJCC-OOTF Size Group in 1416 Patients with Retinoblastoma by Treatment Modality							

			AJCC-OOTF Size Group				
			1: < 50% Globe Involved	2: > 50% and < 2/3 Globe Involved	3: > 2/3 of Globe Involved	4: Diffuse Infiltrating Retinoblastoma	Total
Treatment modality	Primary enucleation	Count	141	175	507	107	930
		% treatment modality	15.2	18.8	54.5	11.5	100.0
		% AJCC-OOTF Size Group	48.8	54.9	75.0	81.1	65.7
		% total	10.0	12.4	35.8	7.6	65.7
	Systemic chemotherapy followed by secondary enucleation	Count	57	55	96	8	216
		% treatment modality	26.4	25.5	44.4	3.7	100.0
		% AJCC-OOTF Size Group	19.7	17.2	14.2	6.1	15.3
		% total	4.0	3.9	6.8	0.6	15.3
	Systemic chemotherapy and eye salvage	Count	91	89	73	17	270
		% treatment modality	33.7	33.0	27.0	6.3	100.0
		% AJCC-OOTF Size Group	31.5	27.9	10.8	12.9	19.1
		% total	6.4	6.3	5.2	1.2	19.1
Total		Count	289	319	676	132	1416
		% total	20.4	22.5	47.7	9.3	100.0

AJCC = American Joint Committee on Cancer; c = clinical; OOTF = Ophthalmic Oncology Task Force; T = tumor.

**Table 3.** Kaplan–Meier Cumulative Proportion of Surviving without Death from Metastasis according to 8th Edition AJCC Clinical cT Subcategory in 1841 Patients with Advanced Retinoblastoma

	Kaplan–Meier Estimate, % (95% CI)		
Variable	*1 yr*	*5 yrs*	*10 yrs*
cT2a (n = 196)	99 (98–100)	98 (97–99)	98 (97–99)
cT2b (n = 814)	98 (97–99)	96 (95–97)	96 (95–97)
cT3a (n = 22)	94 (89–99)	88 (80–96)	88 (80–96)
cT3b (n = 166)	95 (93–97)	95 (93–97)	95 (93–97)
cT3c (n = 389)	94 (89–99)	92 (90–94)	92 (90–94)
cT3d (n = 203)	85 (82–88)	84 (81–87)	84 (81–87)
cT3e (n = 51)	78 (72–84)	75 (68–82)	75 (68–82)

Pairwise Comparisons [Log-Rank Test]						

	*cT2b*	*cT3a*	*cT3b*	*cT3c*	*cT3d*	*cT3e*
cT2a	0.27	0.008	0.047	0.007	<0.001*	<0.001*
cT2b		0.065	0.19	0.002*	<0.001*	<0.001*
cT3a			0.46	0.62	0.46	0.14
cT3b				0.53	0.004	<0.001*
cT3c					0.001*	<0.001*
cT3d						0.127

Kaplan–Meier Cumulative Proportion of Surviving without Death from Metastasis according to Treatment Modality and Tumor Laterality^†^ in 1841 Patients with Advanced Retinoblastoma				

		*Kaplan–Meier Point Estimates (95% CI), %*		
*Variable*		*1 yr*	*5 yrs*	*10 yrs*
Primary enucleation (n = 1128)		96 (95–97)	96 (95–97)	96 (95–97)
	• Unilateral (n = 870)	97 (96–98)	96 (95–97)	96 (95–97)
	• Bilateral (n = 258)	95 (94–96)	94 (92–96)	94 (92–96)
Systemic chemotherapy	Secondary enucleation (n = 315)	93 (91–95)	89 (87–91)	89 (87–91)
	• Unilateral (n = 161)	89 (86–92)	86 (83–89)	86 (83–89)
	• Bilateral (n = 154)	97 (96–98)	93 (91–95)	93 (91–95)
	Eye salvage (n = 398)	94 (93–95)	90 (88–92)	90 (88–92)
	• Unilateral (n = 180)	94 (93–95)	93 (91–95)	93 (91–95)
	• Bilateral (n = 218)	91 (89–93)	88 (85–91)	88 (85–91)

Pairwise Comparisons [Log-Rank]		

	*Systemic Chemotherapy followed by Secondary Enucleation*	*Systemic Chemotherapy and Eye Salvage*
Primary enucleation	<0.001*	<0.001*
Systemic chemotherapy followed by secondary enucleation		0.951

Pairwise Comparisons [Log-Rank]					

	*Primary Enucleation Bilateral*	*Systemic Chemotherapy and Secondary Enucleation Unilateral*	*Systemic Chemotherapy and Secondary Enucleation Bilateral*	*Systemic Chemotherapy and Eye Salvage Unilateral*	*Systemic Chemotherapy and Eye Salvage Bilateral*
Primary enucleation unilateral	0.210	<0.001*	0.327	0.070	<0.001*
Primary enucleation bilateral		0.007	0.905	0.477	0.049
Systemic chemotherapy and secondary enucleation unilateral			0.015	0.094	0.453
Systemic chemotherapy and secondary enucleation bilateral				0.488	0.084
Systemic chemotherapy and eye salvage unilateral					0.284

Overall comparison, *P* < 0.001.

AJCC = American Joint Committee on Cancer; c = clinical; CI = confidence interval; T = tumor.

* Significant after adjustment for multiple comparisons according to Bonferroni.

† The treatment modality for worse eye in bilateral RB cases was attributed to the patient.

**Table 4.** Cox Proportional Hazards Regression Model for 8th Edition AJCC cT Subcategories, Age at Presentation, Treatment Modality, and Heritable Trait Associated with Metastatic Mortality in 1841 Patients with Retinoblastoma

Variable	Patients, No. (N = 1841)	Reference	HR (95% CI)	*P* Value
cT2b	814	cT2a	1.6 (0.5–5.4)	0.424
cT3b	166	cT2a	3.5 (0.9–14.2)	0.082
cT3c	389	cT2a	4.9 (1.4–16.3)	0.011
cT3d	203	cT2a	14.0 (4.2–46.1)	<0.001
cT3e	51	cT2a	19.6 (5.4–71.0)	<0.001
Systemic chemotherapy followed by secondary enucleation	315	Primary enucleation	3.3 (2.0–5.4)	<0.001
Systemic chemotherapy and eye salvage	398	Primary enucleation	4.9 (2.9–8.3)	<0.001
Age (mos)				
8.0–17.0	437	<8.0	0.7 (0.3–1.6)	0.443
17.0–29.0	502	<8.0	2.7 (1.4–5.2)	0.002
>29.0	519	<8.0	2.8 (1.5–5.6)	0.002
H1	636	H0	1.4 (0.9–2.2)	0.116

AJCC = American Joint Committee on Cancer; c = clinical; CI = confidence interval; HR = hazard ratio; T = tumor.

**Table 5.** Kaplan–Meier Cumulative Proportion of Surviving without Metastatic Death for AJCC-OOTF Size Groups in 1416 Patients with Retinoblastoma

		Kaplan–Meier Estimates, % (95% CI)		
Size Group	Variable	*1 yr*	*5 yrs*	*10 yrs*
1, n = 289	< 50% globe involved	100	99 (98–100)	99 (98–100)
2, n = 319	> 50% and < 2/3 globe involved	97 (96–98)	96 (95–97)	96 (95–97)
3, n = 676	> 2/3 of globe involved	95 (94–96)	94 (93–95)	93 (92–94)
4, n = 132	diffuse infiltrating retinoblastoma	84 (81–87)	83 (79–87)	83 (79–87)

Pairwise Comparisons [Log-Rank Test]				

*Size Group*	*2*	*3*	*4*
1	0.035	0.001*	<0.001*
2		0.10	<0.001*
3			<0.001*

Overall comparison: *P* < 0.001.

AJCC = American Joint Committee on Cancer; CI = confidence interval; OOTF = Ophthalmic Oncology Task Force.

* Significant after adjustment for multiple comparisons according to Bonferroni.

**Table 6.** Cox Proportional Hazards Regression Model for AJCC-OOTF Size Groups, Treatment Modality, Age at Presentation, and Heritable Trait Associated with Metastatic Death in 1416 Patients with Retinoblastoma

Variable	Patients, n (%) (n = 1416)	Reference	HR (95% CI)	*P* Value
Size Group 2	319	Size Group 1	4.6 (0.9–21.4)	0.051
Size Group 3	676	Size Group 1	10.0 (2.4–42.2)	0.002
Size Group 4	132	Size Group 1	41.1 (9.4–179.3)	<0.001
Systemic chemotherapy followed by secondary enucleation	216	Primary enucleation	4.3 (2.4–8.1)	<0.001
Systemic chemotherapy and eye salvage	270	Primary enucleation	5.0 (2.6–9.5)	<0.001
Age (mos)				
8.0–17.0	314	< 8.0	0.6 (0.2–2.0)	0.459
17.0–29.0	419	< 8.0	2.5 (1.1–5.5)	0.032
> 29.0	450	< 8.0	3.7 (1.6–8.6)	0.003
H1	296	H0	1.9 (0.9–3.5)	0.052

AJCC = American Joint Committee on Cancer; CI = confidence interval; HR = hazard ratio; OOTF = Ophthalmic Oncology Task Force.

## Abbreviations and Acronyms:

AJCC: American Joint Committee on Cancer
CHLA: Children’s Hospital of Los Angeles
HR: hazard ratio
IQR: interquartile range
OOTF: Ophthalmic Oncology Task Force
RB: retinoblastoma
SD: standard deviation
WEH: Wills Eye Hospital

## References

[1] Finger PT. Do you speak ocular tumor? Ophthalmology. 2003;110:13–14. 12511339 10.1016/s0161-6420(02)01838-9

[2] Finger PT. Foundational elements for collaboration in ophthalmic oncology. Ophthalmol Retina. 2017;1:263–265. 31047507 10.1016/j.oret.2017.01.002

[3] Kletke SN, Feng ZX, Hazrati LN, Clinical predictors at diagnosis of low-risk histopathology in unilateral advanced retinoblastoma. Ophthalmology. 2019;126:1306–1314. 30986443 10.1016/j.ophtha.2019.04.003

[4] Munier FL, Beck-Popovic M, Chantada GL, Conservative management of retinoblastoma: challenging orthodoxy without compromising the state of metastatic grace. “Alive, with good vision and no comorbidity.”. Prog Retin Eye Res. 2019;73: 100764. 31173880 10.1016/j.preteyeres.2019.05.005

[5] Lumbroso-Le Rouic L, Aerts I, Hajage D, Conservative treatment of retinoblastoma: a prospective phase II randomized trial of neoadjuvant chemotherapy followed by local treatments and chemothermotherapy. Eye Lond Engl. 2016;30:46–52. 10.1038/eye.2015.179 PMC4709527 26427984

[6] Chantada GL, Gonzalez A, Fandino A, Some clinical findings at presentation can predict high-risk pathology features in unilateral retinoblastoma. J Pediatr Hematol Oncol. 2009;31:325–329. 19415010 10.1097/MPH.0b013e3181923cc5

[7] Kashyap S, Meel R, Pushker N, Clinical predictors of high risk histopathology in retinoblastoma. Pediatr Blood Cancer. 2012;58:356–361. 21721113 10.1002/pbc.23239

[8] Kaliki S, Srinivasan V, Gupta A, Clinical features predictive of high-risk retinoblastoma in 403 Asian Indian patients: a case-control study. Ophthalmology. 2015;122:1165–1172. 25841975 10.1016/j.ophtha.2015.01.018

[9] Kim ME, Shah S, Zolfaghari E, An intraocular pressure predictive of high-risk histopathologic features in group e retinoblastoma eyes. Int Ophthalmol Clin. 2019;59:77–86. 30908281 10.1097/IIO.0000000000000265 PMC6681901

[10] Finger PT, Harbour JW, Karcioglu ZA. Risk factors for metastasis in retinoblastoma. Surv Ophthalmol. 2002;47:1–16. 11801265 10.1016/s0039-6257(01)00279-x

[11] Finger PT, Meskin SW, Wisnicki HJ, High-frequency ultrasound of anterior segment retinoblastoma. Am J Ophthalmol. 2004;137:944–946. 15126167 10.1016/j.ajo.2003.10.042

[12] Mallipatna A, Gallie BL, Chévez-Barrios P, Retinoblastoma. In: Amin MB, Edge SB, Greene FL, eds. AJCC Cancer Staging Manual. 8th ed. New York, NY: Springer; 2017:819–831.

[13] College of American Pathologists. Protocol for the Examination of Specimens From Patients With Retinoblastoma. Version: Retinoblastoma 4.1.0.0. CAP; 2021. https://documents.cap.org/protocols/Retinoblastoma_4.1.0.0.REL_CAPCP.pdf. Accessed January 30, 2022.

[14] Finger PT. Eye: Choroidal melanoma, retinoblastoma, ocular adnexal lymphoma and eyelid cancers. In: O’Sullivan B (Editor in Chief), Brierley J, D’Cruz A, , eds. Union For International Cancer Control (UICC) UICC Manual of Clinical Oncology, 9th ed. Hoboken, NJ: John Wiley and Sons, Ltd.; 2015:726–744.

[15] Tomar AS, Finger PT, Gallie B, A multicenter, international collaborative study for AJCC-staging of retinoblastoma: Part I: Metastasis-associated mortality. Ophthalmology. 2020;127:1719–1732. 32512116 10.1016/j.ophtha.2020.05.050

[16] Tomar AS, Finger P, Gallie BL, A multicenter, international collaborative study for AJCC-staging of retinoblastoma: Part II: Treatment success and globe salvage. Ophthalmology. 2020;127:1733–1746. 32526306 10.1016/j.ophtha.2020.05.051

[17] Linn Murphree A Intraocular retinoblastoma: the case for a new group classification. Ophthalmol Clin N Am. 2005;18: 41–53. viii. 10.1016/j.ohc.2004.11.003 15763190

[18] Shields CL, Mashayekhi A, Au AK, The International Classification of Retinoblastoma predicts chemoreducftion success. Ophthalmology. 2006;113:2276–2280. 16996605 10.1016/j.ophtha.2006.06.018

[19] Ophthalmic Oncology Task Force. Retinoblastoma. In: Edge SE, Bryd DR, Compton CA, , eds. AJCC Cancer Staging Manual. 7th ed. New York, NY: Springer; 2010: 561–568.

[20] Scelfo C, Francis JH, Khetan V, An international survey of classification and treatment choices for group D retinoblastoma. Int J Ophthalmol. 2017;10:961–967. 28730089 10.18240/ijo.2017.06.20 PMC5515152

[21] Kim JW, Shah SN, Green S, Tumour size criteria for Group D and E eyes in the International Classification System for Retinoblastoma: effects on rates of globe salvage and high-risk histopathologic features. Acta Ophthalmol (Copenh). 2020;98:e121–e125. 10.1111/aos.14222 PMC7448756 31421017

[22] Tomar AS, Finger PT, Gallie B, Global retinoblastoma treatment outcomes: association with national income level. Ophthalmology. 2021;128:740–753. 33007338 10.1016/j.ophtha.2020.09.032

[23] Global Retinoblastoma Study Group, Fabian ID, Abdallah E, Global retinoblastoma presentation and analysis by national income level. JAMA Oncol. 2020;6:685–695. 32105305 10.1001/jamaoncol.2019.6716 PMC7047856

[24] Traine PG, Schedler KJ, Rodrigues EB. Clinical presentation and genetic paradigm of diffuse infiltrating retinoblastoma: a review. Ocul Oncol Pathol. 2016;2:128–132. 27239450 10.1159/000441528 PMC4881270

[25] Schofield PB. Diffuse infiltrating retinoblastoma. Br J Ophthalmol. 1960;44:35–41. 14443414 10.1136/bjo.44.1.35 PMC513741

[26] Tomar AS, Finger PT, Gallie BL. High-risk pathology by presenting features in advanced intraocular retinoblastoma: a multicenter, international data-sharing AJCC Study. Ophthalmology. 2022;S0161-6420(22):00284–00286. 10.1016/j.ophtha.2022.04.006 PMC9329269 35436535

[27] Finger PT, Tomar AS. Retinoblastoma outcomes: a global perspective. Lancet Glob Health. 2022;10:e307–e308. 35093203 10.1016/S2214-109X(21)00598-2

[28] Bowman RJC, Mafwiri M, Luthert P, Outcome of retinoblastoma in east Africa. Pediatr Blood Cancer. 2008;50: 160–162. 17120241 10.1002/pbc.21080

[29] Aschero R, Francis JH, Ganiewich D, Recurrent somatic chromosomal abnormalities in relapsed extraocular retinoblastoma. Cancers. 2021;13:673. 33567541 10.3390/cancers13040673 PMC7915502

