# Supplementary figures and images for: Metastatic Death Based on Presenting Features and Treatment for Advanced Intraocular Retinoblastoma: A Multicenter Registry-Based Study
Source: Ophthalmology. Author manuscript; Available in PMC 2022 Aug 1. (PMC9329221; doi:10.1016/j.ophtha.2022.04.022)

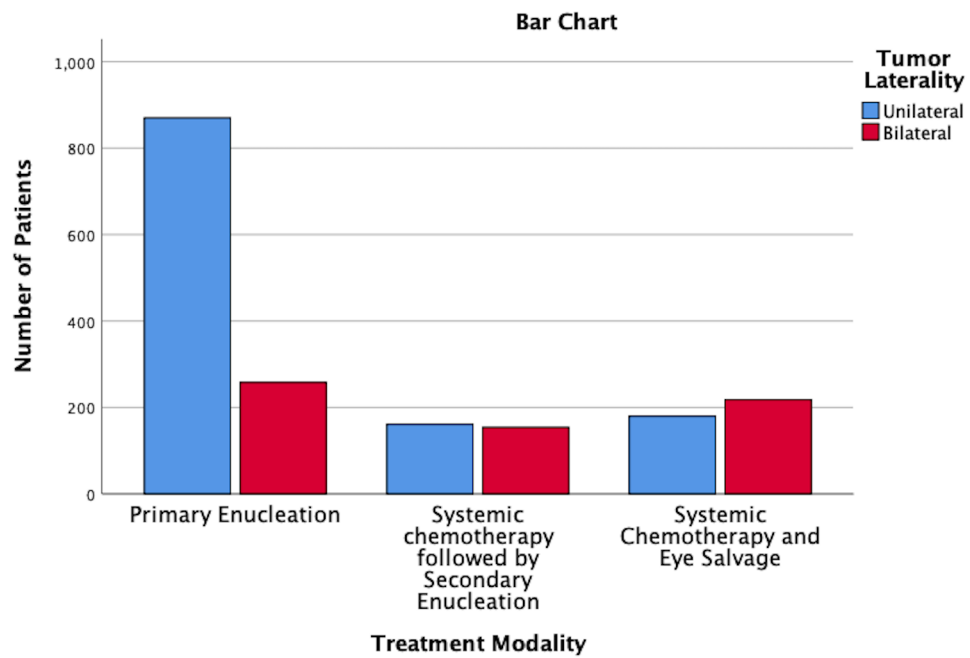

Supplement: sup fig 1 [file NIHMS1816535-supplement-sup_fig_1.pdf]
