# Supplementary material for: Metastatic Death Based on Presenting Features and Treatment for Advanced Intraocular Retinoblastoma: A Multicenter Registry-Based Study
Source: Ophthalmology. Author manuscript; Available in PMC 2022 Aug 1. (PMC9329221; doi:10.1016/j.ophtha.2022.04.022)
Supplement: sup fig 2 [file NIHMS1816535-supplement-sup_fig_2.pdf]

Cumulative Survival of Advanced Retinoblastoma Patients Based on Treatment Modalities and Tumor Laterality

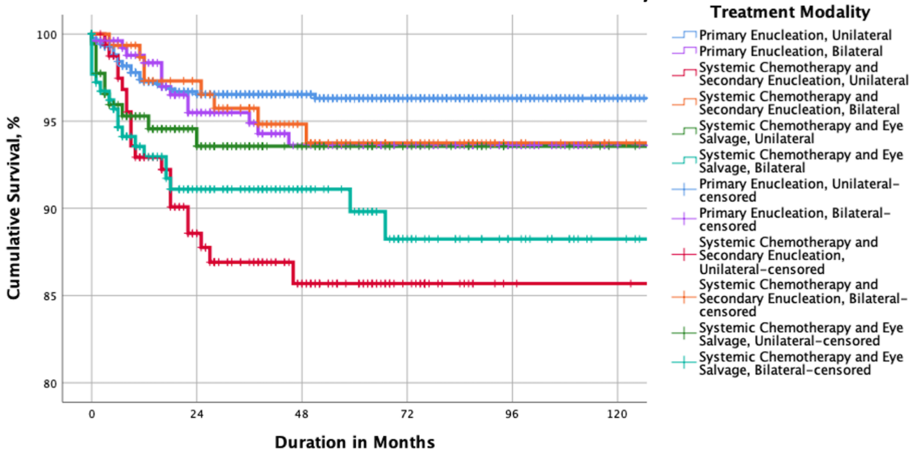

Numbers at risk

Primary Enucleation	Unilateral	870	627	446	290	167	79
	Bilateral	258	183	137	98	56	24
Systemic Chemotherapy & secondary enucleation	Unilateral	161	111	68	29	10	9
	Bilateral	154	127	87	54	27	17
Systemic Chemotherapy and eye salvage	Unilateral	180	95	48	23	10	7
	Bilateral	218	130	82	46	30	18
