# Supplementary material for: Metastatic Death Based on Presenting Features and Treatment for Advanced Intraocular Retinoblastoma: A Multicenter Registry-Based Study
Source: Ophthalmology. Author manuscript; Available in PMC 2022 Aug 1. (PMC9329221; doi:10.1016/j.ophtha.2022.04.022)
Supplement: sup fig 3 [file NIHMS1816535-supplement-sup_fig_3.pdf]

Cumulative Survival of Advanced Retinoblastoma Patients Based on AJCC Clinical T Sub-category

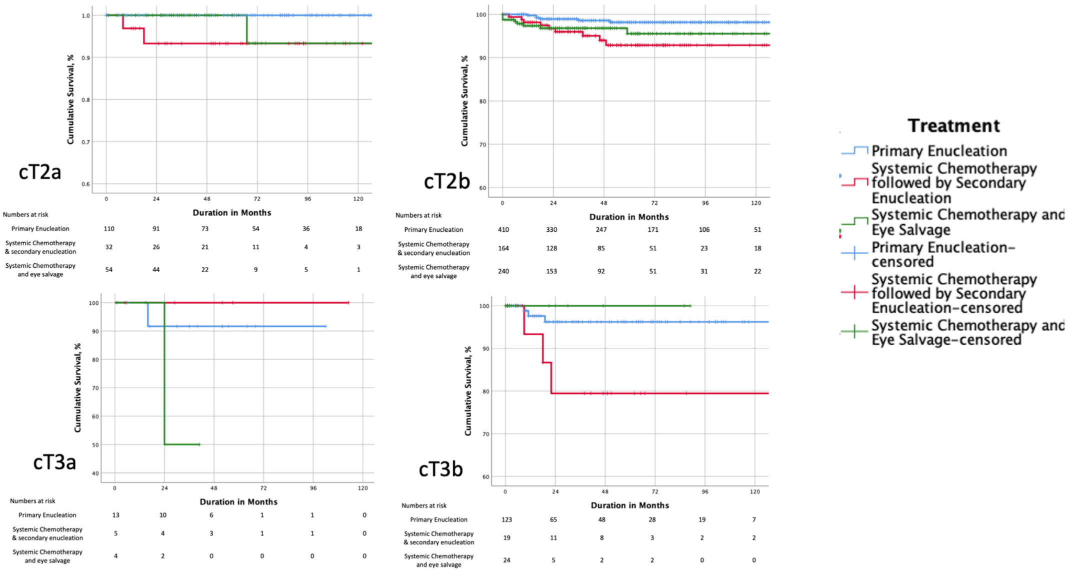

cT2a

Numbers at risk	0	24	48	72	96	120
Primary Enucleation	110	91	73	54	36	18
Systemic Chemotherapy & secondary enucleation	32	26	21	11	4	3
Systemic Chemotherapy and eye salvage	54	44	22	9	5	1

cT2b

Numbers at risk	0	24	48	72	96	120
Primary Enucleation	410	330	247	171	106	51
Systemic Chemotherapy & secondary enucleation	164	128	85	51	23	18
Systemic Chemotherapy and eye salvage	240	153	92	51	31	22

cT3a

Numbers at risk	0	24	48	72	96	120
Primary Enucleation	13	10	6	1	1	0
Systemic Chemotherapy & secondary enucleation	5	4	3	1	1	0
Systemic Chemotherapy and eye salvage	4	2	0	0	0	0

cT3b

Numbers at risk	0	24	48	72	96	120
Primary Enucleation	123	65	48	28	19	7
Systemic Chemotherapy & secondary enucleation	19	11	8	3	2	2
Systemic Chemotherapy and eye salvage	24	5	2	2	0	0
