# Supplementary material for: Metastatic Death Based on Presenting Features and Treatment for Advanced Intraocular Retinoblastoma: A Multicenter Registry-Based Study
Source: Ophthalmology. Author manuscript; Available in PMC 2022 Aug 1. (PMC9329221; doi:10.1016/j.ophtha.2022.04.022)

Cumulative Survival of Advanced Retinoblastoma Patients Based on AJCC Clinical T Sub-category

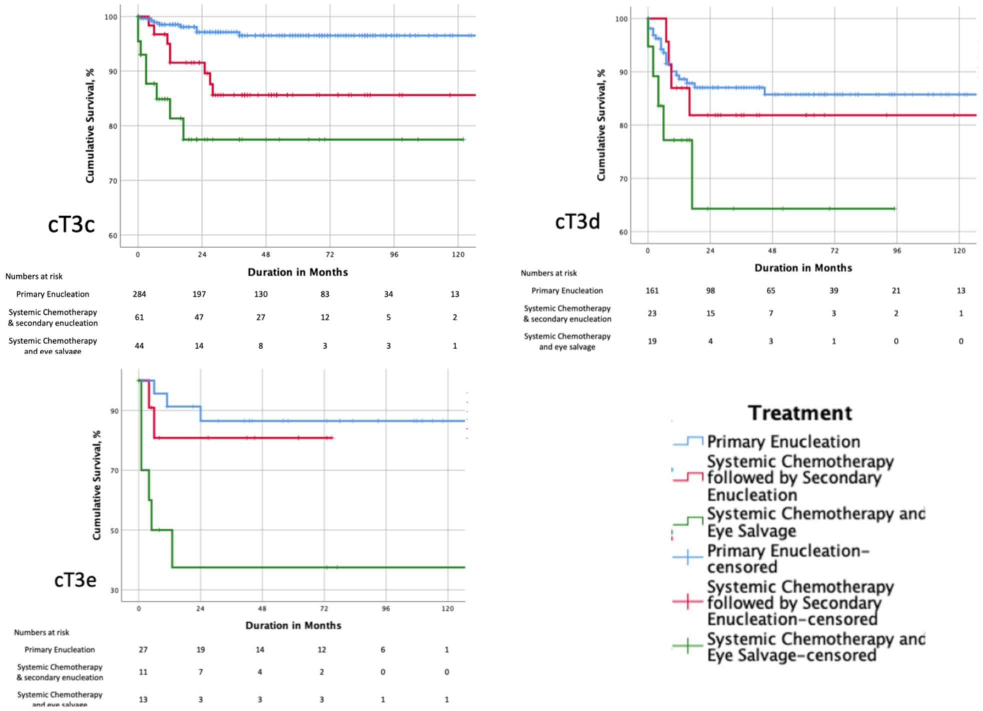

Supplement: sup fig 4 [file NIHMS1816535-supplement-sup_fig_4.pdf]
