# Supplementary material for: Metastatic Death Based on Presenting Features and Treatment for Advanced Intraocular Retinoblastoma: A Multicenter Registry-Based Study
Source: Ophthalmology. Author manuscript; Available in PMC 2022 Aug 1. (PMC9329221; doi:10.1016/j.ophtha.2022.04.022)
Supplement: supp fig 5 [file NIHMS1816535-supplement-supp_fig_5.pdf]

Cumulative Survival of Advanced Retinoblastoma Patients Based on AJCC OOTF Size Groups and Tumor Laterality

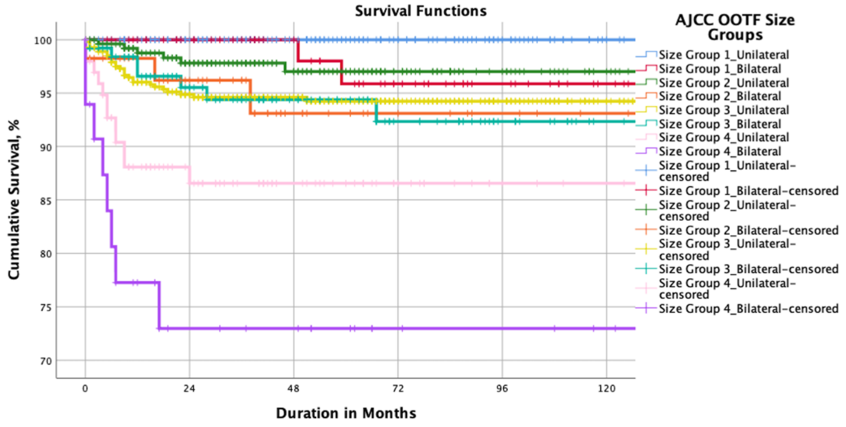

Numbers at risk

Size Group 1	Unilateral	216	159	111	75	44	24
	Bilateral	73	62	51	37	25	16
Size Group 2	Unilateral	262	192	122	69	41	24
	Bilateral	57	40	22	14	10	7
Size Group 3	Unilateral	549	376	266	161	84	36
	Bilateral	127	89	60	37	17	5
Size Group 4	Unilateral	99	58	34	19	10	6
	Bilateral	33	15	13	6	5	3
