# Supplementary material for: Metastatic Death Based on Presenting Features and Treatment for Advanced Intraocular Retinoblastoma: A Multicenter Registry-Based Study
Source: Ophthalmology. Author manuscript; Available in PMC 2022 Aug 1. (PMC9329221; doi:10.1016/j.ophtha.2022.04.022)

Cumulative Survival of Advanced Retinoblastoma Patients Based on AJCC-OOTF Size Groups and Treatment Modalities

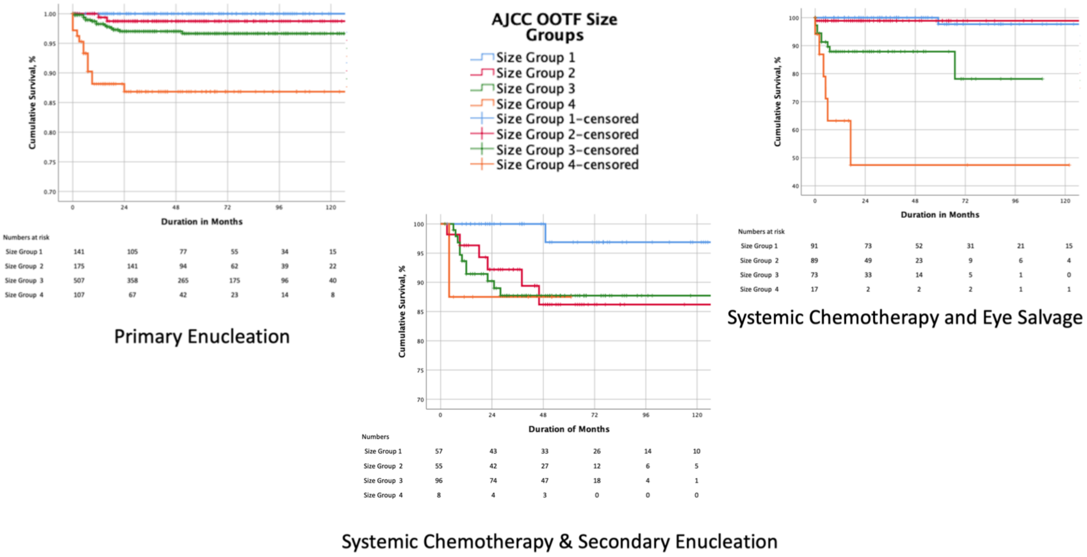

Supplement: supp fig 6 [file NIHMS1816535-supplement-supp_fig_6.pdf]
