# Supplementary material for: Metastatic Death Based on Presenting Features and Treatment for Advanced Intraocular Retinoblastoma: A Multicenter Registry-Based Study
Source: Ophthalmology. Author manuscript; Available in PMC 2022 Aug 1. (PMC9329221; doi:10.1016/j.ophtha.2022.04.022)
Supplement: sup tab 1 [file NIHMS1816535-supplement-sup_tab_1.pdf]

Supplementary Table 1: Patient-level (Worse Eye) Tumor Laterality in 1841 Patients with Retinoblastoma and Treatment Modality

			Tumor Laterality		Total
			Unilateral	Bilateral	
Treatment Modality	Primary Enucleation	Count	870	258	1128
		% Treatment Modality	77.1%	22.9%	100.0%
		% Laterality	71.8%	41.0%	61.3%
		% Total	47.3%	14.0%	61.3%
	Systemic Chemotherapy followed by secondary enucleation	Count	161	154	315
		% Treatment Modality	51.1%	48.9%	100.0%
		% Laterality	13.3%	24.4%	17.1%
		% Total	8.7%	8.4%	17.1%
	Systemic Chemotherapy and eye salvage	Count	180	218	398
		% Treatment Modality	45.2%	54.8%	100.0%
		% Laterality	14.9%	34.6%	21.6%
		% Total	9.8%	11.8%	21.6%
Total		Count	1211	630	1841
		% Total	65.8%	34.2%	100.0%

A chi-square test was performed to examine the relation between tumor laterality and preferred treatment modality. The relation between these variables was significant, $\chi(2) = 169.334$, p = <0.001. Unilateral advanced RB are more likely to be primarily enucleated than bilateral tumors.

AJCC: American Joint Committee on Cancer; OOTF: Ophthalmic Oncology Task Force
