# Supplementary material for: Metastatic Death Based on Presenting Features and Treatment for Advanced Intraocular Retinoblastoma: A Multicenter Registry-Based Study
Source: Ophthalmology. Author manuscript; Available in PMC 2022 Aug 1. (PMC9329221; doi:10.1016/j.ophtha.2022.04.022)
Supplement: sup tab 2 [file NIHMS1816535-supplement-sup_tab_2.pdf]

Supplementary Table 2: Kaplan-Meier Cumulative Proportion of Surviving without Metastatic Death according to 8th Edition AJCC Clinical cT subcategory and Treatment Modality

cT2a (N=196)

		Kaplan-Meier Point Estimates (95% CI), %		
Variable		1 year	5 year	10 year
Primary Enucleation (n=110)		100	100	100
Systemic Chemotherapy	Secondary Enucleation (n=32)	94 (90-98)	94 (90-98)	94 (90-98)
	Eye Salvage (n=54)	100	93 (86-100)	93 (86-100)

Overall Comparison p= 0.013

Pairwise comparisons [Log Rank]

	Primary Enucleation	Systemic Chemotherapy followed by secondary enucleation	Systemic Chemotherapy and eye salvage
Primary Enucleation		0.009*	0.047
Systemic Chemotherapy followed by secondary enucleation			0.354

* Significant after adjustment for multiple comparisons according to Bonferroni

cT2b (N=814)

		Kaplan-Meier Point Estimates (95% CI), %		
Variable		1 year	5 year	10 year
Primary Enucleation (n=410)		99 (98-100)	98 (97-99)	98 (97-99)
Systemic Chemotherapy	Secondary Enucleation (n=164)	98 (97-99)	93 (91-95)	93 (91-95)
	Eye Salvage (n=240)	97 (96-98)	96 (94-98)	96 (94-98)

Overall Comparison p= 0.025

Pairwise comparisons [Log Rank]

	Primary Enucleation	Systemic Chemotherapy followed by	Systemic Chemotherapy and eye salvage

		secondary enucleation	
Primary Enucleation		0.005*	0.056
Systemic Chemotherapy followed by secondary enucleation			0.472

* Significant after adjustment for multiple comparisons according to Bonferroni

cT3a (Phthisis, N=22)

		Kaplan-Meier Point Estimates (95% CI), %		
Variable		1 year	5 year	10 year
Primary Enucleation (n=13)		91 (83-99)	91 (83-99)	N/A
Systemic Chemotherapy	Secondary Enucleation (n=5)	100	100	N/A
	Eye Salvage (n=4)	100	N/A	N/A

Overall Comparison p=0.382

cT3b (Anterior chamber involvement, N=166)

		Kaplan-Meier Point Estimates (95% CI), %		
Variable		1 year	5 year	10 year
Primary Enucleation (n=123)		97 (95-99)	97 (95-99)	97 (95-99)
Systemic Chemotherapy	Secondary Enucleation (n=19)	80 (70-90)	80 (70-90)	80 (70-90)
	Eye Salvage (n=24)	100	100	Data N/A

Overall Comparison p=0.029

Pairwise comparisons [Log Rank]

	Primary Enucleation	Systemic Chemotherapy followed by secondary enucleation	Systemic Chemotherapy and eye salvage
Primary Enucleation		0.017*	0.632
Systemic Chemotherapy followed by secondary enucleation			0.264

* Significant after adjustment for multiple comparisons according to Bonferroni

cT3c (Glaucoma, N=389)

		Kaplan-Meier Point Estimates (95% CI), %		
Variable		1 year	5 year	10 year
Primary Enucleation (n=284)		97 (96-98)	97 (96-98)	97 (96-98)
Systemic Chemotherapy	Secondary Enucleation (n=61)	91 (87-93)	85 (80-90)	85 (80-90)
	Eye Salvage (n=44)	75 (67-83)	75 (67-83)	75 (67-83)

Overall Comparison p<0.001

Pairwise comparisons [Log Rank]

	Primary Enucleation	Systemic Chemotherapy followed by secondary enucleation	Systemic Chemotherapy and eye salvage
Primary Enucleation		0.001*	P<0.001*
Systemic Chemotherapy followed by secondary enucleation			0.126

* Significant after adjustment for multiple comparisons according to Bonferroni

cT3d (Intraocular hemorrhage, N=203)

		Kaplan-Meier Point Estimates (95% CI), %		
Variable		1 year	5 year	10 year
Primary Enucleation (n=161)		87 (84-90)	86 (83-89)	86 (83-89)
Systemic Chemotherapy	Secondary Enucleation (n=23)	82 (74-88)	82 (74-88)	82 (74-88)
	Eye Salvage (n=19)	66 (53-79)	66 (53-79)	N/A

Overall Comparison p=0.147

cT3e (Orbital cellulitis, N=51)

		Kaplan-Meier Point Estimates (95% CI), %		
Variable		1 year	5 year	10 year
Primary Enucleation (n=27)		92 (86-98)	87 (80-94)	87 (80-94)
Systemic Chemotherapy	Secondary Enucleation (n=11)	80 (67-93)	80 (67-93)	NA
	Eye Salvage (n=13)	41 (25-57)	41 (25-57)	41 (25-57)

Overall Comparison p=0.004

Pairwise comparisons [Log Rank]

	Primary Enucleation	Systemic Chemotherapy followed by secondary enucleation	Systemic Chemotherapy and eye salvage
Primary Enucleation		0.537	0.001*
Systemic Chemotherapy followed by secondary enucleation			0.045

* Significant after adjustment for multiple comparisons according to Bonferroni

AJCC: American Joint Committee on Cancer; CI: Confidence Interval
