# Supplementary material for: Metastatic Death Based on Presenting Features and Treatment for Advanced Intraocular Retinoblastoma: A Multicenter Registry-Based Study
Source: Ophthalmology. Author manuscript; Available in PMC 2022 Aug 1. (PMC9329221; doi:10.1016/j.ophtha.2022.04.022)
Supplement: sup tab 3 [file NIHMS1816535-supplement-sup_tab_3.pdf]

Supplementary Table 3: Kaplan-Meier Cumulative Proportion of Surviving without Metastatic Death for AJCC-OOTF Size Groups in 1416 Patients with Retinoblastoma

Size Group	Variable	Laterality	Kaplan-Meier estimates, % (95% Confidence Interval)		
			1 year	5 year	10 year
1	< 50% globe involved	Unilateral n= 216	100	100	100
		Bilateral n= 73	100	96 (93-99)	96 (93-99)
2	> 50% and < 2/3 globe involved	Unilateral n= 262	98 (97-99)	97 (96-98)	97 (96-98)
		Bilateral n= 57	98 (96-100)	93 (89-97)	93 (89-97)
3	> 2/3 of globe involved	Unilateral n= 549	96 (95-97)	94 (93-95)	94 (93-95)
		Bilateral n= 127	95 (93-97)	92 (89-95)	92 (89-95)
4	diffuse infiltrating retinoblastoma	Unilateral n= 99	88 (85-91)	86 (82-90)	86 (82-90)
		Bilateral n= 33	73 (65-81)	73 (65-81)	73 (65-81)

Overall comparison: $p < 0.001$

Pairwise comparisons [Log Rank test]

Size Group	Unilateral vs Bilateral
1	0.027
2	0.227
3	0.908
4	0.072

AJCC: American Joint Committee on Cancer; OOTF: Ophthalmic Oncology Task Force
