# Supplementary material for: Metastatic Death Based on Presenting Features and Treatment for Advanced Intraocular Retinoblastoma: A Multicenter Registry-Based Study
Source: Ophthalmology. Author manuscript; Available in PMC 2022 Aug 1. (PMC9329221; doi:10.1016/j.ophtha.2022.04.022)
Supplement: sup tab 4 [file NIHMS1816535-supplement-sup_tab_4.pdf]

Supplementary Table 4: Kaplan-Meier Cumulative Proportion of Surviving without Metastatic Death for AJCC-OOTF Size Groups and Treatment Modality in Patients with Retinoblastoma

Primary Enucleation (N=930)

Variable		Kaplan-Meier estimates, % (95% Confidence Interval)		
Size Group		1 year	5 year	10 year
1<br>n= 141	< 50% globe involved	100	100	100
2<br>n= 175	> 50% and < 2/3 globe involved	99 (98-100)	99 (98-100)	99 (98-100)
3<br>n= 507	> 2/3 of globe involved	97 (96-98)	97 (96-98)	97 (96-98)
4<br>n= 107	diffuse infiltrating retinoblastoma	88 (85-91)	87 (84-90)	87 (84-90)

Overall comparison: p< 0.001

Pairwise comparisons [Log Rank test]

Size Group	2	3	4
1	0.219	0.047	<0.001*
2		0.190	<0.001*
3			<0.001*

* Significant after adjustment for multiple comparisons according to Bonferroni

Systemic Chemotherapy followed by Secondary Enucleation (N= 216)

Variable		Kaplan-Meier estimates, % (95% Confidence Interval)		
Size Group		1 year	5 year	10 year
1<br>n= 57	< 50% globe involved	100	97 (94-100)	97 (94-100)
2<br>n= 55	> 50% and < 2/3 globe involved	92 (88-96)	86 (81-91)	86 (81-91)
3<br>n= 96	> 2/3 of globe involved	90 (87-93)	87 (83-91)	87 (83-91)
4<br>n= 8	diffuse infiltrating retinoblastoma	86 (73-99)	86 (73-99)	N/A

Overall comparison: p=0.114

Systemic Chemotherapy and Eye Salvage (N= 270)

Variable		Kaplan-Meier estimates, % (95% Confidence Interval)		
Size Group		1 year	5 year	10 year
1<br>n= 91	< 50% globe involved	100	98 (96-100)	98 (96-100)
2<br>n= 89	> 50% and < 2/3 globe involved	99 (98-100)	99 (98-100)	99 (98-100)
3<br>n= 73	> 2/3 of globe involved	87 (83-91)	76 (65-87)	N/A

4<br>n= 17	diffuse infiltrating retinoblastoma	52 (37-67)	52 (37-67)	52 (37-67)

Overall comparison: $p < 0.001$

Pairwise comparisons [Log Rank test]

Size Group	2	3	4
1	0.770	0.001*	<0.001*
2		0.003*	<0.001*
3			0.011

* Significant after adjustment for multiple comparisons according to Bonferroni

AJCC: American Joint Committee on Cancer; OOTF: Ophthalmic Oncology Task Force
