# Supplementary material for: Metastatic Death Based on Presenting Features and Treatment for Advanced Intraocular Retinoblastoma: A Multicenter Registry-Based Study
Source: Ophthalmology. Author manuscript; Available in PMC 2022 Aug 1. (PMC9329221; doi:10.1016/j.ophtha.2022.04.022)
Supplement: supp data [file NIHMS1816535-supplement-supp_data.pdf]

Study group list

American Joint Committee on Cancer Ophthalmic Oncology Task Force members: Paul T. Finger, MD (Chair) (New York Eye Cancer Center, New York); Sarah E. Coupland, MBBS, PhD, FRCPath (Vice Chair) (Royal Liverpool University Hospital, Liverpool, United Kingdom); Daniel M. Albert, MD, MS (Oregon Health and Sciences University, Portland); Anush G. Amiryan, MD, and Svetlana Saakyan, MD (Moscow Helmholtz Research Institute of Eye Disease, Moscow, Russian Federation); Claudia Auw-Hädrich, MD (University of Freiburg, Freiburg, Germany); Diane Baker, CTR (American Joint Committee on Cancer, Chicago, Illinois); Raymond Barnhill, MD, MSc (UCLA Medical Center, Los Angeles); José M. Caminal, MD, PhD (Bellvitge University Hospital, Hospitalet de Llobregat, Barcelona, Spain); William L. Caroll, MD (New York University Cancer Institute–New York University Langone Medical, New York); Nathalie Cassoux, MD, PhD, Laurence G. Desjardins, MD, François Doz, MD, MSc, and Gaelle Pierron, PhD (Institut Curie, Paris, France); Jaume Catalá-Mora, MD (SJD Barcelona Children's Hospital, Hospital Sant Joan de Déu, Barcelona, Spain); Guillermo Chantada, MD (Hospital JP Garrahan, Buenos Aires, Argentina); Patricia Chévez-Barrios, MD (Houston Methodist Hospital, Houston, Texas); R. Max Conway, MD, PhD (Save Sight Institute, Sydney, Australia); Bertil E. Damato, MD, PhD (University of California, San Francisco, San Francisco); Hakan Demirci, MD (Kellogg Eye Center, Ann Arbor, Michigan); Jonathan J. Dutton, MD, PhD (University of North Carolina, Chapel Hill); Bita Esmaeli, MD, Victor G. Prieto, MD, PhD, and Michelle Williams, MD (University of Texas MD Anderson Cancer Center, Houston); Brenda L. Gallie, MD (Hospital for Sick Children, Toronto, Ontario, Canada); Gerardo F. Graue, MD (Instituto de Oftalmología Fundación Conde de Valenciana, Mexico City,

Mexico); Hans E. Grossniklaus, MD (Emory Eye Center, Atlanta, Georgia); Steffen Heegaard, MD, PhD (University of Copenhagen, Glostrup Hospital, Copenhagen, Denmark); Leonard M. Holbach, MD (University Erlangen, Nürnberg, Germany); Santosh G. Honavar, MD (Centre For Sight Super-Specialty Eye Hospital, Hyderabad, India); Martine J. Jager, MD, PhD (Leiden University Medical Center, Leiden, the Netherlands); Tero Kivelä, MD, FEBO, and Emma Kujala, MD (Helsinki University Hospital, Helsinki, Finland); Livia Lumbroso-Le Rouic, MD (Institute Claudius Regaud Medical Center, Toulouse, France); Ashwin C. Mallipatna, MBBS, MS, DNB (Hospital for Sick Children, Toronto, Canada); Giulio M. Modorati, MD (San Raffaele Hospital, Milan, Italy); Francis L. Munier, MD (Jules-Gonin Eye Hospital, Lausanne, Switzerland); Timothy G. Murray, MD, MBA (Murray Ocular Oncology and Retina, Coral Gables, Florida); Anna C. Pavlick, MD (New York University Cancer Center, New York); Jacob Pe’er, MD (Hebrew University, Hadassah Ein Karem Hospital, Jerusalem, Israel); David E. Pelayes, MD (Buenos Aires University, Buenos Aires, Argentina); Manuel Jorge Rodriguez, MD (Mayo Clinic, Jacksonville, Florida); Wolfgang A.G. Sauerwein, MD, PhD (University Hospital Essen, Essen, Germany); Ekaterina Semenova, MD (The New York Eye and Ear Infirmary of Mount Sinai, New York); Stefan Seregard, MD (St Erik’s Eye Hospital, Karolinska Institute, Stockholm, Sweden); Carol Shields, MD (Wills Eye Institute, Philadelphia, Pennsylvania); E. Rand Simpson, MD, FRCS(C) (Mount Sinai Hospital, Toronto, Ontario, Canada); Arun D. Singh, MD (Cole Eye Institute, Cleveland Clinic, Cleveland, Ohio); Shigenobu Suzuki, MD, PhD (National Cancer Center Hospital, Tokyo, Japan); Mary Kay Washington, MD, PhD (Vanderbilt University Medical Center, Nashville, Tennessee); Valerie A. White, MD, MHSc, FRCPC (Vancouver Coastal Health Research Institute, Vancouver, British Columbia, Canada); Mathew W. Wilson, MD (University of Tennessee Health Science Center, Memphis); Christian

W. Wittekind, MD (Institut fur Pathologie der Universität, Leipzig, Germany); and Vivian Yin, MPH (Memorial Sloan Kettering Cancer Center, New York, New York).
